# Hepatocyte miR‐33a mediates mitochondrial dysfunction and hepatosteatosis by suppressing *NDUFA5*

**DOI:** 10.1111/jcmm.13918

**Published:** 2018-10-16

**Authors:** Hezhongrong Nie, Xiaohong Yu, Haihong He, Lintao Zhou, Qing Li, Chunli Song, Damin Wang, Tingyu Ren, Zeyan Chen, Hanlian Huang, Xiaoyan Dai, Yiwen Zhou

**Affiliations:** ^1^ Center of Clinical Laboratory Shenzhen Hospital Southern Medical University Shenzhen China

**Keywords:** hepatocyte, lipid, liver, microRNA‐33a, mitochondria, NDUFA5

## Abstract

Emerging evidence suggests that microRNAs (miRNAs) are essential for metabolic haemostasis of liver tissues. Among them, miR‐33a is supposed to modulate the cholesterol export and fatty acid oxidation, but whether miR‐33a involves in the process of fatty liver disease is unclear. To disclose the hypothesis, we utilized miR‐33a mimic and antisense to explore their effects in primary hepatocytes or high‐fat diet (HFD)‐fed mice. Treatment with palmitic acid (PA) or HFD significantly increased the expression of miR‐33a in hepatocytes or liver tissues. In primary hepatocytes, miR‐33a mimic decreased mitochondrial function, including reduction of ATP production and oxygen consumption, whereas miR‐33a inhibition protected PA‐induced mitochondrial dysfunction. Interestingly, miR‐33a selectively suppressed mitochondrial complex I activity and protein expression, but not other complexes. Through bioinformatics prediction, we found miR‐33a directly targeted on the 3′‐UTR of *NDUFA5*. Dual‐luciferase reporter analysis further confirmed the direct suppression of miR‐33a on *NDUFA5* expression. More importantly, administration of miR‐33a antisense could effectively restore HFD‐induced mitochondrial dysfunction through up‐regulation of *NDUFA5* levels. Mice treated with miR‐33a antisense also exhibited improved liver function and structural disorders under obese status. Taken together, miR‐33a was an important mediator of hepatocyte mitochondrial function, and the therapeutic benefits implied miR‐33a antisense had the potential clinical application in combating the fatty liver disease.

## 1 INTRODUCTION

Long‐term overnutrition primarily leads individuals to unhealthy status with metabolic disorders.^1^ Among them, hepatic steatosis is the most common metabolic complication in clinical practice, affecting most obese individuals.^2^, ^3^ Because of possessing various vital functions, liver tissues require highly orchestrated and controlled biochemical processes. Increasing evidence suggests that microRNA (miRNA), a short noncoding RNA molecule of 18‐25 nucleotides in length, is essential for the regulation of liver development, metabolic functions and injuries.^4^, ^5^, ^6^ Multiple miRNAs have been implicated in liver diseases through repressing specific hepatic functional mRNAs by degradation or translational repression.^4^, ^5^, ^6^ Hence, alterations in intrahepatic miRNA have been associated with liver disease, and might be potential therapeutic targets to combat liver disease.

Evidence indicated that there was obvious altered expression of miRNA profiles in the fatty liver, as compared with normal liver. A microarray analysis of human fatty liver identified 23 differentially expressed miRNAs, among which miR‐122 and miR‐126 were down‐regulated and miR‐34a, miR‐21 and miR‐100 were up‐regulated.^7^ Similarly, a mouse study explored the expression profiles of miRNAs in long‐term diet‐induced fatty liver showed a total of nine up‐regulated and 18 down‐regulated miRNAs.^8^ Our previous study demonstrated that hepatocyte miR‐194 mediated diet‐induced fatty liver disease.^9^ Among them, miR‐33a was identified as an important miRNA in regulating liver function.^10^ MiR‐33a inhibited Sirtuin 6 to enhance triglyceride synthesis in the mouse liver, whereas inhibition of endogenous miR‐33a led to a significant increase in cholesterol export.^11^, ^12^ However, whether miR‐33a involves in the process of fatty liver disease is unknown.

As a fundamental metabolic tissue, liver controls energy haemostasis of whole body. Stored lipids are consumed and synthesized to triacylglycerol, phospholipids, and/or cholesterol esters in hepatocytes.^4^ Among these process, hepatic mitochondria orchestrates energy metabolism by controlling the β‐oxidation, tricarboxylic acid cycle (TCA), adenosine triphosphate (ATP) synthesis through oxidative phosphorylation (OXPHOS) and then releasing reactive oxygen species (ROS).^13^, ^14^ Mitochondrial dysfunction is involved in the development of fatty liver disease.^13^, ^14^ Therefore, mitochondrial activity is closely associated with hepatic energy homoeostasis. Previous study also showed miR‐33a could modulate the mitochondrial function in macrophages.^15^ Inhibition of miR‐33a could effectively increase the activity of AMPK, a key regulatory enzyme that acts to promote ATP‐generating pathways.^16^ However, the direct targets of miR‐33a in the mitochondrial function is not clear.

In present study, we targeted to investigate the role of miR‐33a in the diet‐induced hepatic dysfunction and mitochondrial disorders. Present results found that increased miR‐33a suppressed mitochondrial complex I activity and the expression of its subunits in hepatocytes. Antisense treatment of miR‐33a attenuated diet‐induced hepatic damages and mitochondrial dysfunction, indicating inhibition of miR‐33a might improve fatty liver disease.

## 2 MATERIALS AND METHODS

### 2.1 Reagents

Lentiviruses encoding mouse miR‐33a mimic, miR‐33a antisense oligonucleotide (ASO) or control vectors were purchased from ThermoFisher Scientific (Waltham, MA). Palmitic acid, bovine serum albumin, haematoxylin, eosin solution and oil red O stain kits were purchased from Sigma chemicals (Louis, MO, USA). Alanine transaminase (ALT), aspartate transaminase (AST) and triglyceride test kits were purchased from Wako (Richmond, VA). Anti‐total OXPHOS, anti‐α‐porin and anti‐NDUFA5 antibodies were purchased from Abcam (Cambridge, MA, USA); anti‐NFUFAF7 antibody was purchased from Sigma chemicals (Louis, MO); anti‐Tubulin was from Cell Signaling (Danvers, MA). ATP measurement kit was purchased from Molecular Probes (Molecular Probes, Carlsbad, USA).

### 2.2 Mouse primary hepatocyte experiment

Mouse primary hepatocytes were isolated from C57BL/6J mice by perfusion with collagenase type IV as previously described.^17^ The palmitic acid (PA) was dissolved in fatty acid‐free bovine serum albumin (BSA) solution, and the final concentration was 100 m mol L^−1^ for storage. For PA treatment, the hepatocytes were stimulated with 100 μ mol L^−1^ PA for 0, 12, 24 and 36‐hour. For miR‐33a mimic treatment, the cells were transfected with 1×10^6 ^IU lentivirus encoding miR‐33a mimic or control vector for 24 hours.

### 2.3 Adenosine triphosphate (ATP) measurement

Primary hepatocyte ATP was measured using an ATP measurement kit (Molecular Probes, Carlsbad) according to our previous published protocols.^18^, ^19^ Briefly, the isolated primary hepatocytes were isolated and washed with cold PBS, and then boiled in 100 μl extraction reagent (100 m mol L^−1^ Tris, 4 m mol L^−1^ EDTA, adjusted to pH 7.75 with acetic acid) for 90 seconds. Supernatants were retrieved by centrifugation at 10 000 *g* for 60 seconds. ATP contents were determined by measuring the luminescence of supernatants mixed with luciferase assay buffer using a Varioskan™ Flash Multimode Reader (Thermo Scientific). ATP luminescence was normalized by protein concentration.

### 2.4 Oxygen consumption

Oxygen consumption in hepatocytes was done according to previous report.^19^ Endogenous basal oxygen consumption was measured with a clark electrode in a water‐jacketed chamber connected to a circulating water bath (Hansatech, Norfolk, UK).

### 2.5 Enzyme activities of OXPHOS complexes

The activity of the electron transfer chain (ETC) complex I (NADH: ubiquinone reductase), II/III (succinate: cytochrome c reductase), IV (cytochrome c oxidase) and V (ATP synthase) were determined according to the method described in previous report.^19^ All ETC activities were expressed as a ratio to citrate synthase activity to account for mitochondrial enrichment.

### 2.6 Animal experiment

Six‐week old, male C57BL/6J mice were used. Mice were fed a chow diet (10 kcal% fat) or a high‐fat diet (HFD, 60 kcal% fat; Research Diets, NJ) for 12‐week. Then, the HFD‐fed mice were tail‐vein injected with lentivirus encoding 1×10^12 ^IU miR‐33a ASO or control vector for single injection. After 4‐week treatment, mice were killed under ether anaesthesia. Hepatic tissues were frozen in liquid nitrogen for analysing gene expression and embedded in 4% paraformaldehyde for structural analysis. In addition, serum was separated and stored in −80°C condition until analysis. Protocols used for all animal studies were approved by the Southern Medical University Animal Policy and Welfare Committee.

### 2.7 Histopathological analysis

After dehydration, the 5‐μm sections were stained with hematoxylin and eosin (H&E) for evaluating the histopathological damage. Besides, the sections were also stained with oil red O staining reagent for evaluating the lipid accumulation. Each image of sections was obtained using a light microscope (Nikon, Japan).

### 2.8 Biochemical analysis

Serum levels of ALT and AST were measured with biochemical reagents according to the manufacturer's instructions.

### 2.9 mRNA analysis

Total RNA was extracted by using TRIzol (Invitrogen, Shanghai), and complementary DNA was synthesized by RNA reverse kit and quantified by RT‐qPCR Kit (Invitrogen, Shanghai). The sequence of primers were listed as following: NDUFA5: F‐ GCCACCACGCTCTTCTGTCTA; R‐GATGAGAGGGAGGCCATTTG. NDUFAF7: F‐TCCATCCAGTTGCCTTCTTG; R‐GGTCTGTTGGGAGTGGTATC. NDUFS5: F‐CCCTTGGGTGTCAAAGGTAA; R‐GCCCTCGCTTATGATCTGTC. GAPDH: F‐AGGAGCGAGACCCCACTAAC; R‐GATGACCCTTTTGGCTCCAC. The relative amount of each gene was normalized to the amount of GAPDH.

### 2.10 Protein analysis

Cell lysate (50 μg) was subjected to 12% SDS‐polyacrylamide gel electrophoresis gel, and transferred onto polyvinyldene fluoride membrane (Bio‐Rad Laboratory, CA). After blocking in 10% milk blocking buffer for 1.5 hours, membranes were incubated with anti‐total OXPHOS, anti‐α‐porin, anti‐NDUFA5, anti‐NDUFAF7 or anti‐Tubulin antibody overnight at 4°C. Then membranes were washed in TBST and incubated with secondary antibody (Cell signaling technology, CA) for 1 hour at room temperature. The protein bands were then visualized using enhanced chemiluminescence reagents (Bio‐Rad, CA).

### 2.11 Statistical analysis

Data were presented as mean ± SD. Student's *t* test was used for comparing two groups, and one‐way ANOVA was used for comparing three or four groups. GraphPad Prism 5 (GraphPad, San Diego, CA) was used to analyse the statistical significance between sets of data. Differences were considered to be significant at *P* < 0.05.

## 3 RESULTS

### 3.1 Palmitic acid or high fat diet up‐regulates the expression of miR‐33a in vitro and in vivo

**Figure 1:**
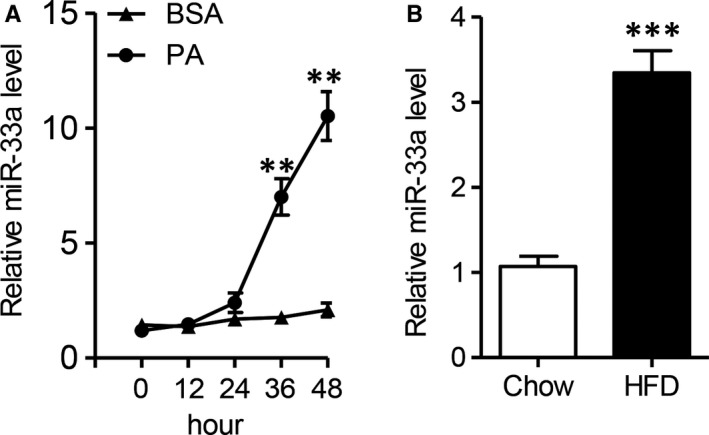
The up‐regulation of hepatocyte miR‐33a level after palmitic acid‐ or high‐fat diet treatment. A, 5 × 10^5^ mouse primary hepatocytes were treated with palmitic acid (PA, 100 μM) for 12, 24, 36 and 48 h, and RT‐qPCR analysis of cellular miR‐33a levels. B, Male C57BL/6J mice were fed with high‐fat diet (HFD) or chow diet (chow) for 12 wk. RT‐qPCR analysis of miR‐33a in liver tissues. Data are shown as mean ± SEM. **P < 0.01, ***P < 0.001, n = 5 independent experiments or 6 mice/group

Previous studies showed miR‐33a was expressed in hepatocytes, and closely associated with liver dysfunction.^10^, ^16^ To address whether miR‐33a also participates the process of diet‐induced hepatic damage, we first treated the primary hepatocytes with palmitic acid (PA), a saturated fatty acid, to investigate the expression of miR‐33a. As Figure 1A shown, PA time‐dependently increased miR‐33a level in hepatocytes. Similarly, high‐fat diet (HFD) also significantly induced the expression of hepatic miR‐33a in mice after HFD for 12 weeks (Figure 1B, *P < *0.001). These results implied hepatocyte miR‐33a was up‐regulated in the overnutrition‐induced liver disease.

### 3.2 Overexpression of miR‐33a decreases mitochondrial activities in hepatocytes

**Figure 2:**
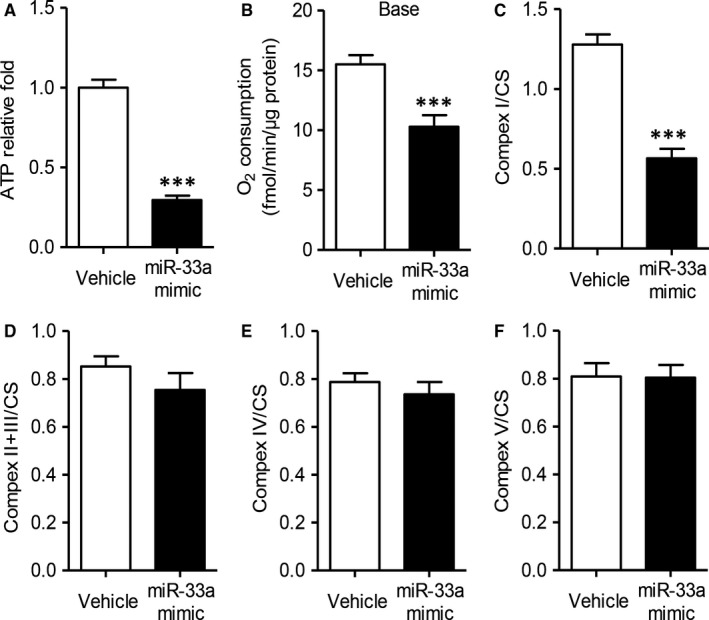
Overexpression of miR‐33a inhibits mitochondrial activity. 5 × 10^5^ mouse primary hepatocytes were transfected with 1 × 10^6 ^IU lentivirus encoding miR‐33a mimic or control vector for 24 h. Biochemical analysis for ATP production (A), basal oxygen consumption (B) and ETC complex I (C), II/III (D), IV (E) and V (F). Data are shown as mean ± SEM. ***P < 0.001 vs vehicle group, n = 5 independent experiments

It is well‐established that hepatic mitochondria orchestrates energy metabolism by substrate oxidation via β‐oxidation, tricarboxylic acid cycle (TCA), adenosine triphosphate (ATP) synthesis through oxidative phosphorylation (OXPHOS).^13^ Mitochondrial ATP synthesis provides 75 % total intracellular ATP, and maintains energy homoeostasis.^13^ Previous studies also found miR‐33a as a critical regulator in mitochondrial metabolism.^15^ Therefore, we next explore the role of miR‐33a in hepatocyte mitochondrial function. The primary hepatocytes were incubated with lentivirus encoding miR‐33a mimic for 24 hours. As Figure 2A shown, mitochondrial ATP level was significantly dropped by 63 % in miR‐33a‐treated hepatocytes (*P < *0.001). The miR‐33a mimic also decreased the basal oxygen consumption by 34 % (Figure 2B, *P < *0.001). To further investigate the respiration properties, we measured the endogenous respiration activity of intact hepatic cells. ATP is synthesized through oxidative phosphorylation, which mainly depends on the activities of the electron transfer chain (ETC) complex.^13^, ^14^ As shown in Figure 2, miR‐33a mimic only decreased ETC complex I activity (Figure 2C, *P < *0.001), but not II/III, IV or V activity (Figure 2D‐F). These results demonstrated miR‐33a lowered hepatocyte mitochondrial activities by affecting complex I.

### 3.3 Treatment with miR‐33a antisense oligonucleotide protects PA‐induced hepatocyte mitochondrial dysfunction

**Figure 3:**
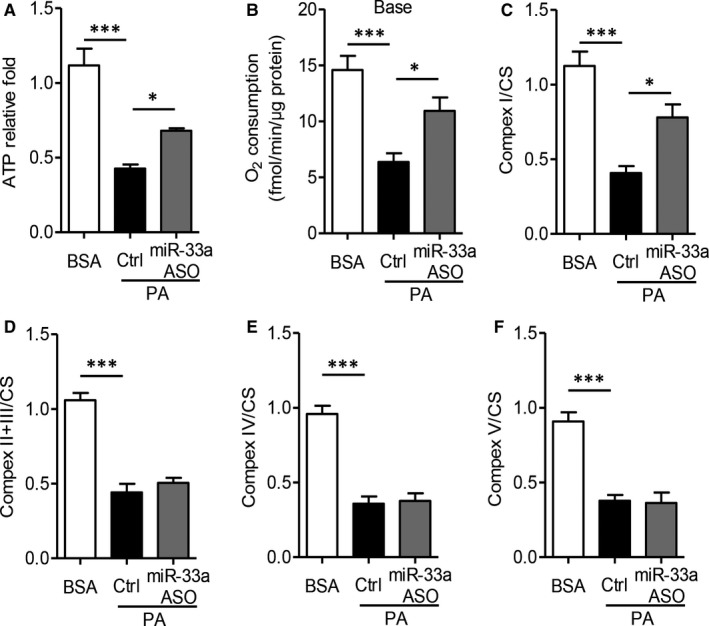
MiR‐33a antisense oligonucleotide inhibited palmitic acid‐induced mitochondrial dysfunction in mouse primary hepatocytes. 5 × 10^5^ mouse primary hepatocytes were transfected with 1 × 10^7^ IU lentivirus encoding miR‐33a ASO or control vector, then stimulated with palmitic acid (PA, 100 μmol L^−1^) for 48 h. Biochemical analysis for ATP production (A), basal oxygen consumption (B) and ETC complex I (C), II/III (D), IV (E) and V (F). Data are shown as mean ± SEM. *P < 0.05, ***P < 0.001, n = 5 independent experiments

Next, we further investigated the effects of palmitic acid (PA)‐induced miR‐33a on hepatocyte mitochondrial function. Previous studies have already demonstrated that palmitic acid obviously disordered mitochondrial function, including reduction of ATP production and complex activities (ref). The primary mouse hepatocytes were treated with miR‐33a inhibitor, an antisense oligonucleotide (ASO) or control antisense, then stimulated with PA for 48 hours. As Figure 3 shown, mitochondrial ATP level was significantly dropped by 66 % in palmitic acid‐treated hepatocytes (*P* < 0.001), but miR‐33a ASO increased ATP production to 1.5‐fold (Figure 3A, *P* < 0.05). The miR‐33a ASO also maintained the basal oxygen consumption (Figure 3B, *P* < 0.05). To further investigate the respiration properties, we measured the endogenous respiration activity of intact hepatic cells. Palmitic acid obviously decreased the activities of all mitochondrial complexes (*P* < 0.001), whereas miR‐33a ASO only reversed ETC complex I activity (Figure 3C, *P* < 0.05), but not II/III, IV or V activity (Figure 3D‐F). These in vitro results further confirmed miR‐33a lowered hepatocyte mitochondrial activities by affecting complex I.

### 3.4 MiR‐33a suppresses mitochondrial complex I subunit NDUFA5

**Figure 4:**
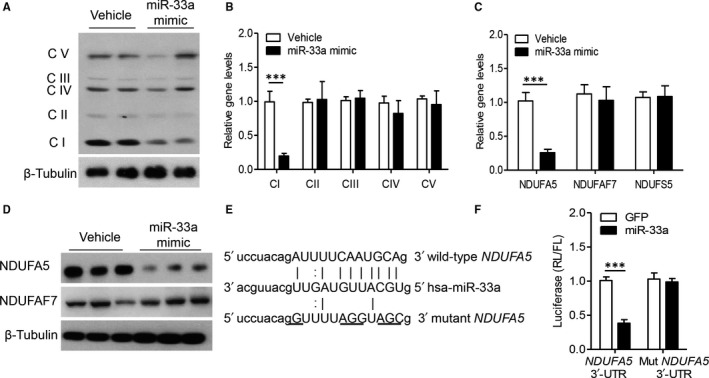
MiR‐33a directly suppresses the expression of mitochondrial complex I subunit *NDUFA5*. 5 × 10^5^ mouse primary hepatocytes were transfected with 1×10^6 ^IU lentivirus encoding miR‐33a mimic or control vector. A‐B. 50 μg total protein was subjected to Western blot (A), and quantitatively analysed the relative expression of mitochondrial complexes (B). C, Total RNA was subjected to real‐time PCR, and the relative levels of *NDUFA5*,* NDUFAF7* and *NDUFS5* were measured. D, Western blot analysis of NDUFA5 and NDUFAF7 levels. E. The conserved binding site on *NDUFA5* mRNA 3′‐UTR for miR‐33a, and mutant sequence for at *NDUFA5* 3′‐UTR. F. The dual‐luciferase reporter assay with plasmids encoding wild‐type *NDUFA5* 3′‐UTR or mutant 3′‐UTR, transfected with miR‐33a overexpression or control plasmid in HEK‐293 cells. Data are shown as mean ± SEM. ***P < 0.001 vs vehicle group, n = 5 independent experiments

Classically, the biological roles of miRNAs are in action through inhibiting the expression of targeting genes. Therefore, we aim to explore how miR‐33a modulates the function of mitochondrial complex I. We first measured the expression of different mitochondrial complexes in primary hepatocytes after treatment with miR‐33a mimic. As shown in Figure 4A,B, miR‐33a mimic selectively suppressed complex I level, but had no obvious effects on other complexes. Bioinformatic analysis indicated miR‐33a might regulate complex I by inhibiting the gene expression of *NDUFA5*,* NDUFAF7* or *NDUFS5*. Intriguingly, miR‐33a significantly decreased *NDUFA5* level by 71 % (Figure 4C, *P* < 0.001), but not changed the gene expression of *NDUFAF7* or *NDUFS5* (Figure 4C). Consistently, miR‐33a obviously decreased protein expression of NDUFA5, but not NDUFAF7 (Figure 4D). More importantly, as Figure 4E shown, *NDUFA5* 3′‐UTR contained the binding sequence of miR‐33a. Therefore, we constructed *NDUFA5* wild‐type and mutant 3′‐UTR sequence, and cotransfected with miR‐33a into HEK‐293T cells. Through luciferase reporter assay, we found that miR‐33a could directly bind to *NDUFA5* 3′‐UTR, but not to mutant *NDUFA5* 3′‐UTR (Figure 4F, *P* < 0.001).

### 3.5 Treatment with miR‐33a antisense oligonucleotide protects HFD‐induced hepatic mitochondrial dysfunction and hepatic injuries

**Figure 5:**
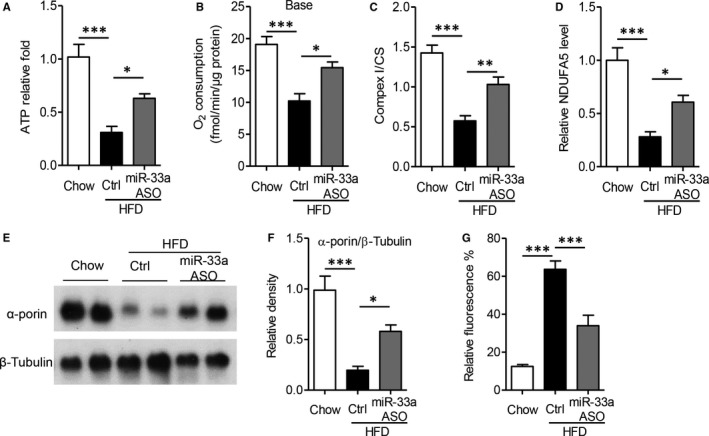
MiR‐33a antisense oligonucleotide protects high‐fat diet‐induced mitochondrial dysfunction. 6‐wk male C57BL/6J mice were fed with high‐fat diet (HFD) for 12 wk, then tail‐vein injected with 1×10^12 ^IU lentivirus encoding miR‐33a antisense oligonucleotide (ASO) or control vector for 4 wk. A‐D. Biochemical analysis for ATP production (A), basal oxygen consumption (B), ETC complex I (C) and *NDUFA5* level (D) in hepatic tissues. E‐F. Western blot analysis of α‐porin and house‐keeping β‐Tubulin (E), and quantitative measurement of α‐porin level (F). G, Fluorescence analysis of reactive oxygen species (ROS) level in hepatic tissues. Data are shown as mean ± SEM. *P < 0.05, **P < 0.01, ***P < 0.001 vs HFD mice treated with control vector, n= 6 mice/group

Finally, we aim to disclose the biological function of miR‐33a in vivo, especially in diet‐induced fatty liver disease. Mice fed with HFD for 12 weeks were tail‐vein injected with miR‐33a inhibitor, an antisense oligonucleotide (ASO) for 4 weeks. As Figure 5A shown, HFD decreased ATP production by 64 %, but miR‐33a ASO significantly increased the ATP levels (*P* < 0.05). Consistent with the findings in primary hepatocytes, HFD largely inhibited the oxygen consumption (Figure 5B), ETC complex I activity (Figure 5C) and *NDUFA5* level (Figure 5D). On the contrast, miR‐33a ASO obviously increased oxygen consumption, complex I activity and *NDUFA5* level in HFD‐fed mouse hepatocyte mitochondria. Consistently, miR‐33a ASO could not reverse the HFD‐down‐regulated activities of complex II, III, IV or V in hepatic tissues (Figure S1).

Dynamic changes in the mitcohindria are key characteristics of mitochondrial dysfunction in metabolic diseases. According to previous studies, there are a set of markers for mitochondrial dynamic changes in fatty liver disease, including ROS generation and mitochondrial mass. In our results, high‐fat diet decreased mitochondrial mass (represented by α‐porin level), as compared with chow diet‐fed mice (Figure 5E,F, *P *< 0.001). However, miR‐33a ASO remarkably increased mitochondrial mass (Figure 5E,F, *P *< 0.05). Meanwhile, the induction of ROS generation in fatty liver was also significantly decreased in miR‐33a ASO‐treated mice (Figure 5G, *P *< 0.001). These results indicated miR‐33a was involved in high‐fat diet‐induced mitochondrial dynamic changes in liver.

**Figure 6:**
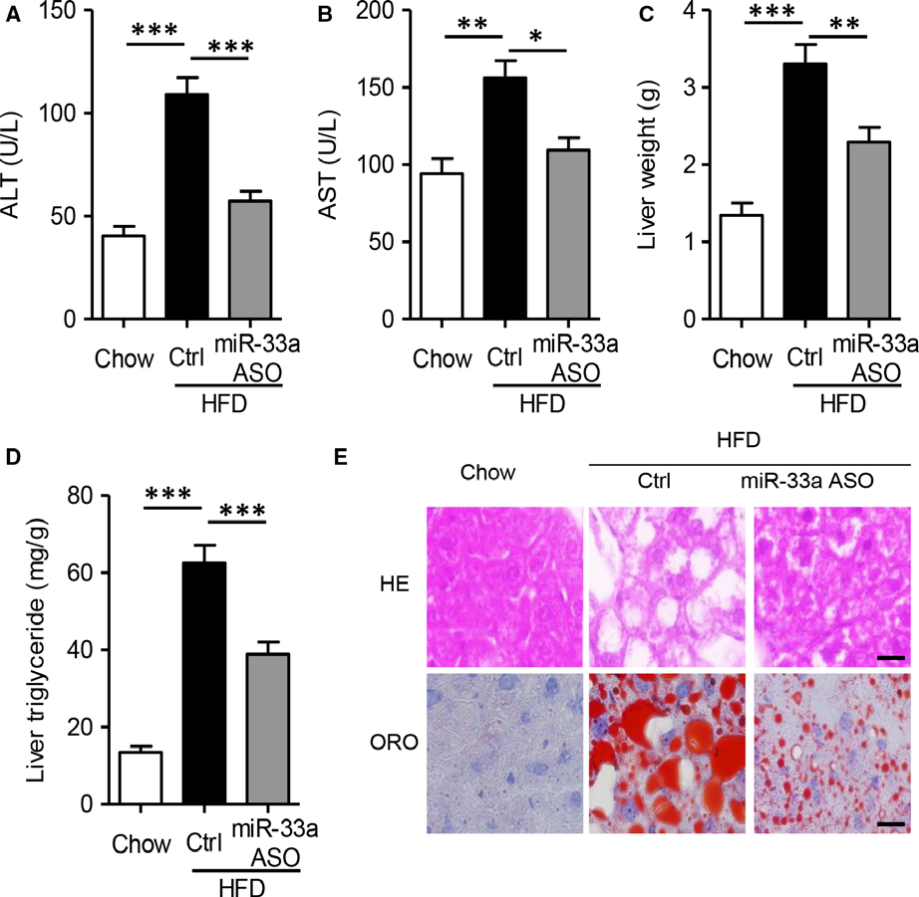
MiR‐33a ASO attenuates HFD‐induced liver injuries. 6‐wk male C57BL/6J mice were fed with high‐fat diet (HFD) for 12 wk, then tail‐vein injected with 1 × 10^12 ^IU lentivirus encoding miR‐33a antisense oligonucleotide (ASO) or control vector for 4 wk. A‐B. Biochemical analysis of serum alanine transaminase (ALT, A) and aspartate transaminase (AST, B). C‐D. Measure the liver weight (C) and hepatic triglyceride levels (D). Data are shown as mean ± SEM. *P < 0.05, **P < 0.01, ***P < 0.001 vs HFD mice treated with control vector, n = 6 mice/group. E. The hepatic slides were subjected to haematoxylin and eosin (H&E) staining (upper panel) and oil red O (ORO) staining (lower panel)

Then, we measured the protective function of miR‐33a ASO in fatty hepatic injuries. Alanine aminotransferase (ALT) and aspartate aminotransferase (AST) are clinical markers for liver damages and diseases, and higher levels of serum ALT and AST indicate liver dysfunction.^20^ As Figure 6A,B shown, HFD feeding induced obviously higher levels of ALT and AST (*P* < 0.01), but treatment with miR‐33a ASO could significantly attenuate these disorders. Both abnormal increase in liver weight and triglycerides were also significantly reversed in miR‐33a ASO‐treated mice, as compared with vehicle‐treated HFD‐fed mice (Figure 6C,D, *P* < 0.01). In the histological analysis showed in Figure 6E, we also found miR‐33a ASO could effectively prevent structure disorders and lipid accumulation, which was illustrated in Oil Red O staining. All these results certainly demonstrated the therapeutic benefits of miR‐33a ASO in ameliorating diet‐induced fatty liver disease.

## 4 DISCUSSION

Hepatic mitochondria control the energy haemostasis and biological function of liver tissues. Abnormal expression or activities of mitochondrial complex initiate the imbalance of energy intake and expenditure, which finally induce hepatic injuries, including lipid accumulation, overactivated inflammatory response and dysfunction. Previous studies indicated miR‐33a might participate in the regulation of hepatic metabolism, therefore the current study aimed to explore the underlying mechanisms and potential therapeutic application. Our findings demonstrated the lipid‐ or diet‐induced miR‐33a not only suppresses mitochondrial complex I activity, but also selectively inhibits the expression of *NDUFA5* in hepatocytes. More importantly, the oligo antisense of miR‐33a could protect diet‐induced fatty liver disease. All these results supported that hepatocyte miR‐33a mediated mitochondrial dysfunction and hepatic injuries by directly targeting the mitochondrial complex I subunit *NDUFA5*.

Many researchers have reported that the aberrant expression of miRNAs in hepatic tissue was related to the pathogenesis of liver disease, including viral hepatitis, hepatocellular carcinoma and fatty liver disease.^7^, ^8^ MiR‐122, the most abundant miRNA in the liver, seems to be an important factor for the metabolism of glucose and lipids.^21^ Additionally, miR‐33a was also found to be a key regulator of such metabolic programs as cholesterol and fatty acid homoeostasis.^11^, ^12^ MiR‐33a is cotranscribed with its host gene endoplasmic reticulum‐bound sterol regulatory element‐binding protein (*Srebp*)‐2, and regulated multiple genes.^11^ The cooperation of miR‐33a and *Srebp‐2* was vital for regulating lipid metabolism within cells. Overexpression of miR‐33a down‐regulated the expression of genes involved in fatty acid metabolism as well as cholesterol biosynthesis, including carnitine O‐octaniltransferase (CROT), carnitine palmitoyltransferase (CPT) 1a, hydroxyacyl‐CoA‐dehydrogenase (HADHB), Sirtuin (SIRT)6, and AMP kinase subunit‐α.^12^ Furthermore, miR‐33a also targeted the insulin receptor substrate 2, an essential component of the insulin‐signalling pathway in the liver.^16^ On the contrast, inhibition of endogenous miR‐33 increased the expression of CROT, CPT1a, HADHB, SIRT6, AMPKα and IRS2 and up‐regulated fatty acid oxidation and insulin signalling.^12^, ^16^ However, the molecular mechanisms underlying the regulation of lipid homoeostasis by miR‐33a are still unclear. Based on the previous findings, the current study first showed that miR‐33a mediated palmitic acid‐ or diet‐induced hepatocyte injuries via suppressing mitochondrial function. Overexpression of miR‐33a inhibited mitochondrial complex I activity and expression (Figures 2and 3), but miR‐33a antisense effectively protected diet‐induced hepatic injuries and mitochondrial dysfunction in obese mice (Figures 4 and 5).

Hepatocytes are rich in mitochondria, which composed by five individual complexes named complex I to V.^13^, ^14^ Mitochondria are the primary cellular organelles for the oxidation and metabolism of fatty acids and glucose, thus mitochondrial dysfunction may contribute to the increase in lipid accumulation.^13^ Furthermore, abnormal mitochondrial activity or expression obviously causes lipid accumulation and increases lipid toxic metabolites which may in turn cause hepatic injuries.^14^ Although accumulating evidence indicated that abnormal mitochondrial homeostasis involved in hepatic injuries, the potential regulators of mitochondrial activity or expression was still unclear. Previous studies found treatment of macrophages with miR‐33a inhibitor led to increased mitochondrial respiration and ATP production.^15^ These findings supported miR‐33 as an important regulator of the availability of fuel sources utilized in mitochondrial respiration. Current study further explored the crosstalk between miR‐33a and mitochondria. As Figure 3 shown, NDUFA5, an important subunit on mitochondrial complex I, was directly targeted by miR‐33a. This finding introduced a novel mechanism to explain the interaction between miR‐33a and mitochondria in hepatocyte.

NDUFA5, as a hydrophobic fraction of the mitochondrial complex I multisubunit enzyme, is believed to be not involved in catalysis, but participated in transferring electrons from NADH to the respiratory chain.^22^, ^23^ The expression of NDUFA5 was reduced in brains of autism patients, which might be attributed to mitochondrial dysfunction and impaired ATP synthesis.^24^ Present study provided evidence that abnormal NDUFA5 expression was involved in the process of fatty liver disease. Therefore, up‐regulation of hepatocyte NDUFA5 level might have benefits in preventing diet‐induced mitochondrial dysfunction and fatty liver disease.

Notably, we firstly explored the pharmacological benefits of miR‐33a antisense oligonucleotide (ASO) in high‐fat diet‐fed mouse model. As shown in Figure 5, miR‐33a ASO reversed diet‐induced mitochondrial dysfunction, including down‐regulation of ATP production (Figure 5A), oxygen consumption (Figure 5B), complex I activity (Figure 5C), *NDUFA5* levels (Figure 5D), mitochondrial mass (Figure 5E‐F) and ROS generation (Figure 5G) in fatty liver. More importantly, miR‐33a ASO also protected hepatic function (Figure 6A,B) and structural haemostasis (Figure 6C‐E). All these results not only supported that miR‐33a was a potential therapeutic target in combating fatty liver diseases, but also demonstrated miR‐33a ASO was possible suitable for clinical applications in the future. In the next research objective, we intend to investigate the clinical correlation between hepatic/circulating miR‐33a levels and hepatoteatosis in human volunteers.

In conclusions, our findings supported that miR‐33a/NDUFA5 axis was an important mediator of hepatocyte mitochondrial function, and the therapeutic benefits implied miR‐33a antisense had the potential clinical application in combating fatty liver disease.

## CONFLICT OF INTEREST

The authors declare that they have no competing interests.

## AUTHOR CONTRIBUTIONS

HZR Nie, XH Yu, HH He, LT Zhou, and HL Huang, Q Li, CL Song, DM Wang and TY Ren performed the animal and cell research; HZR Nie, ZY Chen and XY Dai contributed to generate virus; HZR Nie and YW Zhou designed the research study; HZR Nie analysed the data; HZR Nie and YW Zhou wrote the paper.
