# Supplementary figures and images for: Hepatocyte miR‐33a mediates mitochondrial dysfunction and hepatosteatosis by suppressing NDUFA5
Source: J Cell Mol Med. 2018 Oct 16;22(12):6285–93. doi: 10.1111/jcmm.13918 (PMC6237601; doi:10.1111/jcmm.13918)

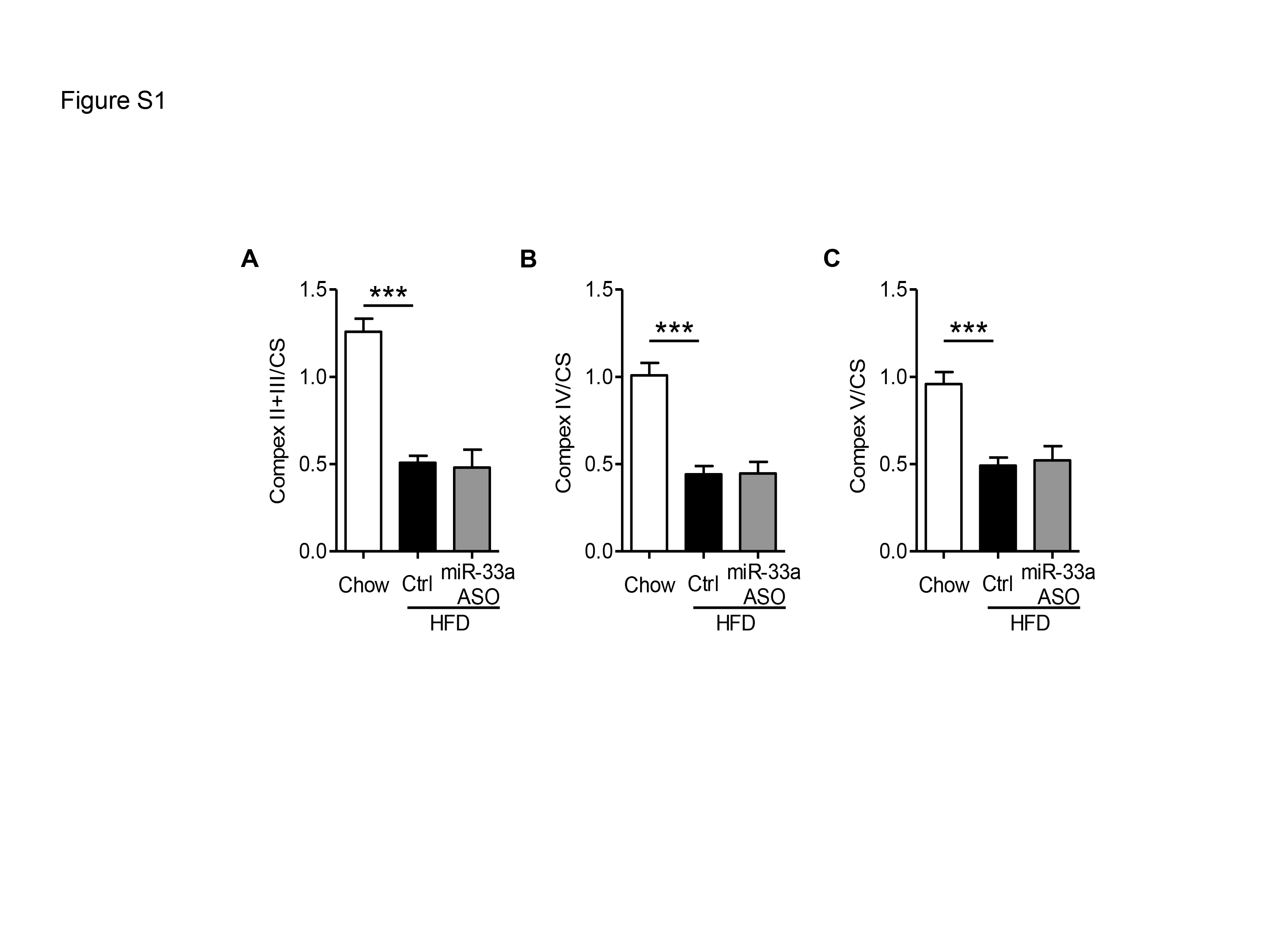

Supplement: Supplementary file 1 [file JCMM-22-6285-s001.tif]
